# Final-year medical students’ perspective: a survey on the use of computed tomography in sepsis

**DOI:** 10.1186/s13244-023-01538-y

**Published:** 2023-11-19

**Authors:** Julian Pohlan, Maria Isabel Opper Hernando, Roderic Waschinsky, Federico Biavati, Harm Peters, Samuel Knauss, Peter Richard Steinhagen, Kerstin Rubarth, Denis Witham, Marc Dewey

**Affiliations:** 1 Department of Radiology, Charité – Universitätsmedizin Berlin, Humboldt-Universität zu Berlin, Freie Universität Berlin, Campus Mitte, Charitéplatz 1, 10117 Berlin, Germany; 2 Berlin Institute of Health at Charité – Universitätsmedizin Berlin, Berlin, Germany; 3 Dieter Scheffner Center for Medical Education, Office of Study Affairs, Charité – Universitätsmedizin Berlin, Campus Mitte, Berlin, Germany; 4 Department of Neurology with Experimental Neurology, Charité – Universitätsmedizin Berlin, Campus Mitte, Berlin, Germany; 5 Department of Gastroenterology and Hepatology, Charité – Universitätsmedizin Berlin, Campus Mitte, Berlin, Germany; 6 Institute for Biometry and Clinical Epidemiology, Charité – Universitätsmedizin Berlin, Campus Mitte, Berlin, Germany; 7 Institute of Medical Informatics, Charité – Universitätsmedizin Berlin, Campus Mitte, Berlin, Germany; 8 Department of Cardiology, Charité – Universitätsmedizin Berlin, Campus Mitte, Berlin, Germany

**Keywords:** Sepsis, Tomography (X-ray computed), Focal infection, Medical students, Survey and questionnaires

## Abstract

**Objectives:**

To determine the perspective of final-year medical students on the use of computed tomography (CT) in patients with sepsis.

**Methods:**

A total of 207 questionnaires were distributed to final-year medical students at a large university medical center, and 113 returned questionnaires met the criteria for inclusion in the analysis. Questions referred to sepsis guidelines, CT indications, and the use of contrast agents. Control variables included a level of practical experience as a final-year student (trimester of student’s practical year) and previous radiological experience. Statistical hypothesis tests such as the Mann-Whitney *U* test and chi-square test were performed.

**Results:**

The majority of participating students, 85% (*n* = 91/107), considered a Systemic Organ Failure Assessment (SOFA) score ≥ 2 as a diagnostic criterion for sepsis. The presence of ≥ 2 positive systemic inflammatory response syndrome (SIRS) criteria was considered relevant for diagnosing sepsis by 34% (*n* = 34/100). Ninety-nine percent (*n* = 64/65) of the participants who fully agreed with a SOFA score ≥ 2 being relevant for diagnosing sepsis would also use it as an indication for a CT scan. Seventy-six percent (*n* = 78/103) of the students rated a known severe allergic reaction to contrast agents as an absolute contraindication for its administration. Ninety-five percent (*n* = 78/82) considered radiation exposure as problematic in CT examinations, especially in repeat CTs.

**Conclusion:**

Most final-year medical students were familiar with the sepsis criteria. Still, some referred to outdated diagnostic criteria. Participants saw the ability to plan further patient management based on CT as a major benefit. Most participants were aware of radiation as a risk of CT.

**Critical relevance statement:**

More detailed knowledge of CT in septic patients should be implemented in the medical curriculum. Retraining of medical students could help increase student confidence potentially improving patient care.

**Key points:**

1. Whereas the majority of final-year medical students were familiar with sepsis criteria, some referred to outdated diagnostic criteria.

2. Participants saw the ability to plan further patient management based on CT as a major benefit.

3. Most participants were aware of radiation as a risk of CT.

**Graphical Abstract:**

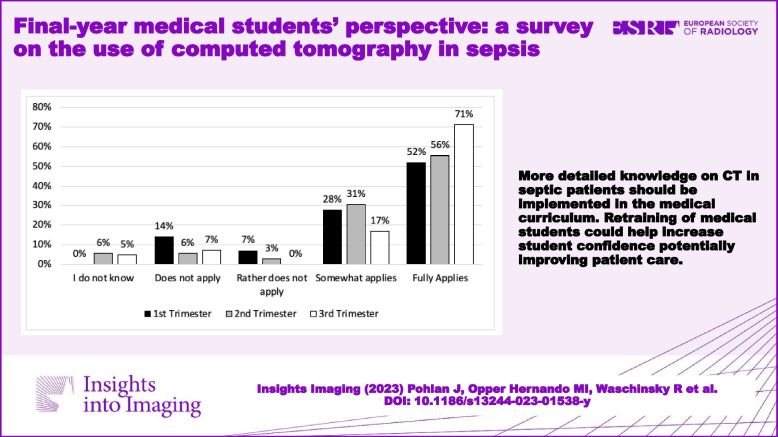

**Supplementary Information:**

The online version contains supplementary material available at 10.1186/s13244-023-01538-y.

## Background

Sepsis is a life-threatening condition with high morbidity, defined as organ dysfunction due to infection [1, 2]. Systemic Organ Failure Assessment (SOFA) score is currently used to diagnose and monitor sepsis, whereas the systemic inflammatory response syndrome (SIRS) criteria were abandoned due to low diagnostic accuracy [2]. Computed tomography (CT) has evolved into an essential diagnostic modality in hospitalized patients, resulting in increased radiation exposure over recent decades [3]. Despite the risks associated with ionizing radiation and administration of intravenous iodine-based contrast agents, CT is considered safe in the appropriate settings [4–6].

International guidelines stress the role of imaging in identifying infectious foci in septic patients [7]. Nevertheless, detailed recommendations on the use of CT are still missing. In surgical patients with sepsis, CT identifies septic foci in more than 50% of the cases [8]. Other studies confirm the value of CT for both diagnostics and therapeutic decision-making [9–11]. Additionally, CT findings may be useful in evaluating the prognosis of septic patients, especially in also ruling out relevant comorbidities [12].

Limited data suggest that medical curricula may not adequately cover aspects of sepsis management [13]. So far, research on medical students primarily focuses on the value of CT in teaching anatomy [14, 15]. One study group has presented data on how to improve the teaching of sepsis-related knowledge in medical students by enriching medical curricula [16]. Another study that investigated an incentive to teach about sepsis did not include the role of CT [17]. Notably, a study examining knowledge in the field of radiology revealed that fourth-year medical students were poorly informed about the crucial aspects of radiology [18]. Overall, the literature on medical students’ knowledge of the application of CT in sepsis is scarce.

The purpose of this study is to investigate final-year medical students’ perspectives—possibly based and derived on teaching content, accumulated experiences, and observations—on the role of CT in patients with sepsis.

## Methods

### Setting

This study was conducted at a large German university clinic. Final-year medical students can come from all German medical faculties—some with a regular, theoretically based medical curriculum and others with a reformed, practice-oriented program. All curricula last a total of 6 years. The last year entails three equally long rotations (trimesters) in internal medicine, surgery, and an elective discipline, and therefore, it is called practical year. The entire year is spent at the hospital in hands-on medical practice. Final-year medical students are supervised by physicians.

### Survey

The authors designed a 12-item questionnaire on sepsis and the use of CT in this indication. The local ethics committee approved the study under the number EA1/203/21.

### Questionnaire structure

The questionnaire was divided into two sections. The first section asked for participants’ demographic data. The second section was divided into three subsections, consisting of questions about sepsis and CT. All questions referred to the scenario of a patient with suspected sepsis.

To determine a level of practical experience as a final-year student, participants indicated in the first section which trimester of their practical year they were in. They were further asked in which areas they had completed previous trimesters and in which fields they had conducted their clinical traineeship. In addition, students were asked about the university they attended before the practical year (if different from the chosen one for the practical year). Lastly, participants had to indicate whether they had undergone any medical training before their studies.

In the second section, we asked participants about their perspectives on diagnostic criteria for sepsis: the SOFA score and the SIRS criteria. For this purpose, a 5-point Likert scale with the following categories was used: *(1) Does not apply*, *(2) Does rather not apply*, *(3) Somewhat applies*, *(4) Fully applies*, and *(5) I do not know*. The following subsection focused on the use of CT in patients with sepsis. The same 5-point Likert scale was utilized to ask about the relevance of clinical parameters for the indication of CT in sepsis. Moreover, in free-text fields, participants could enter possible disadvantages of repeated CT examinations and give suggestions for improving the radiology department’s diagnostic and/or interventional pathways in care for patients with sepsis. We regarded “radiation” and “radiation dose” as synonyms for radiation exposure. Participants were asked how they would proceed if an initial CT fails to detect a focus of infection. For this purpose, they were presented with a list of possibilities for further action. Similarly, by utilizing a list of options, students were asked to indicate what they see as a major benefit of CT examinations in patients with sepsis. Both questions were conducted through a 4-point Likert scale: *(1) Does not apply*, *(2) Does rather not apply*, *(3) Somewhat applies*, and *(4) Fully applies*. Subsequently, participants were asked to indicate on a 4-point Likert scale {*(1) Unenhanced examination*, *(2) Rather unenhanced examination*, *(3) Rather intravenous contrast agent administration*, and *(4) definitive intravenous contrast agent administration*} for which body region to be examined in septic patients they saw an indication for the use of contrast agent. Finally, participants had to state their view on possible contraindications to the administration of contrast agent in patients with sepsis and additional medical problems using the following 4-point Likert scale: *(1) Absolute contraindication for contrast agent administration*, *(2) Relative contraindication*, *(3) Examination with contrast agent possible after appropriate preparation*, and *(4) No contraindication for contrast agent administration*.

### Administration and data handling

**Fig. 1:**
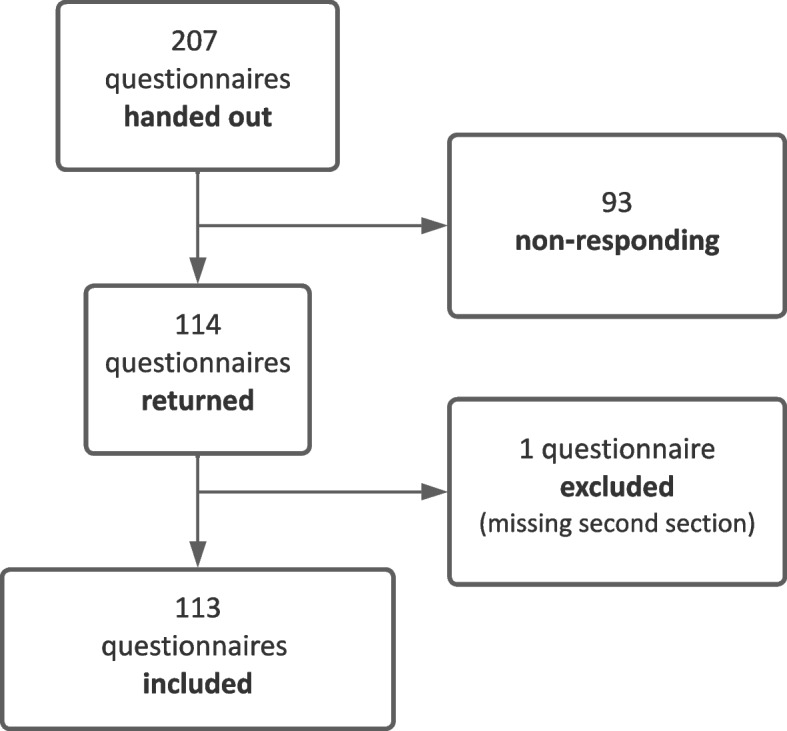
Flowchart of the survey. One hundred fourteen of 207 questionnaires were returned. One questionnaire had to be excluded since the second section of the questionnaire was left blank. The remaining 113 questionnaires were included in the analysis of different question categories. SOFA, Systemic Organ Failure Assessment Score; SIRS, Systemic Inflammatory Response Syndrome Score

Between the 23 August and 31 December 2021, the authors distributed 207 questionnaires to final-year medical students at a German university clinic (Fig. 1). Questionnaires were distributed at their workplace, i.e., in clinical ward or radiology department, for voluntary participation in physicians’ duty rooms. The authors collected 114 answered questionnaires at another time in the same location they were distributed (response rate 55%). The inclusion criteria for analysis were (a) no missing answers in the first section of the questionnaire and (b) complete answers to at least one part of the questions of the second section. Questionnaires with missing answers in the subsections required for analysis were excluded resulting in different numbers of total cases used for the hypothesis analysis.

### Data analysis

The answers given in the questionnaires were extracted manually and collected in Excel tables (Microsoft® Excel® for Microsoft 365 MSO, version 2112, 2017; Microsoft, Redmond, WA, USA). Excel also was used for designing graphs and calculating descriptive statistics such as absolute and relative frequencies. Different numbers of cases were used for the analysis of individual categories. Further statistical hypothesis tests such as the Mann-Whitney *U* test and chi-square test were performed with SPSS (Statistical Package for the Social Sciences, IBM® SPSS Statistics, version 28.0.1.0, 2021, IBM, Armonk, NY, USA). Throughout the paper, percentages have been rounded and provided without decimals. The significance level was set to *α* < 0.05. The *p* values were interpreted as exploratory rather than confirmatory due to the study’s exploratory nature.

## Results

### Study population

Of 113 study participants included in the analysis, 52% (*n* = 59/113) reported their gender as male, 44% (*n* = 50/113) as female, and 4% (*n* = 4/113) as diverse. The average age of the participants was 26.1 years (standard deviation = 2.7). Fifty-six percent (*n* = 63/113) of the participants were matriculated to the same and 44% (*n* = 50/113) to another university (external students) prior to their practical year. Of the external students, 74% (*n* = 37/50) stated to have undergone the regular curriculum (Additional file 1: Table S1). Thirteen percent (*n* = 15/113) of the participants had completed a clinical traineeship or a trimester of their practical year in radiology in advance. With 38% (*n* = 43/113), most participants were in the third trimester of their practical year (Table 1).

**Table 1:** Overview: demographic data

		*n* = total	Relative amount
Attended university prior to the final year, *n* = 113	*Internal*	63	56%
	*External*	50	44%
Study program, *n* = 113	*Reformed curriculum*	76	67%
	*Regular curriculum*	37	33%
Level of practical experience as a final-year medical student, *n* = 113	*1st trimester*	30	27%
	*2nd trimester*	40	35%
	*3rd trimester*	43	38%
Previous radiological experience, *n* = 25	*Past trimester/internship in radiology*	15	60%
	*Emergency training*	10	40%

### Understanding of sepsis diagnosis and perspective on the use of CT

**Fig. 2:**
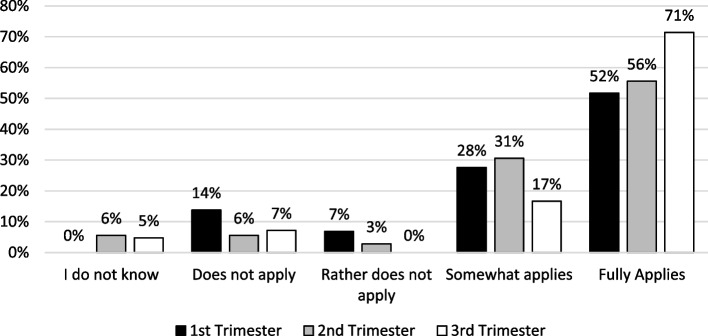
Participant answers to “SOFA score ≥ 2 is a diagnostic criterion for sepsis” sorted by current trimester. The option *(4) Fully applies* was selected by the majority of participants. Fifty-two percent (*n* = 15/29) of first-trimester students, 56% (*n* = 20/36) of second-trimester students, and 71% (*n* = 30/42) of third-trimester students saw a SOFA score ≥ 2 as a diagnostic criterion for sepsis, followed by 17% (*n* = 7/42) of 3rd, 31% (*n* = 11/36) of 2nd, and 28% (*n* = 8/29) of 1st-trimester students somewhat agreeing. The group most likely to disagree were the 1st-trimester students (21%, *n* = 6/29). Overall, 11% (*n* = 12/107) of all participants chose the option “I do not know.” Total *n* = 107/113; missing responses in 6 participants. SOFA, Systemic Organ Failure Assessment; SOFA score ≥ 2, SOFA score greater than or equal to two points

A SOFA score greater than or equal to two points (SOFA score ≥ 2) was considered relevant for diagnosing sepsis by 85% (*n* = 91/107) of the participants. Of those, 71% (*n* = 65/91) fully agreed and 29% (*n* = 26/91) somewhat agreed. Students of all three levels of practical experience as a final-year student mostly considered a SOFA score ≥ 2 a diagnostic criterion for sepsis (Fig. 2). Most likely to fully disagree were students from the first trimester (14%, *n* = 4/29). The differences between the first- and third-trimester students were not significant (*p* = 0.14, chi-square test). Across all levels of practical experience, a SOFA score ≥ 2 as a reason for requesting a CT scan was most frequently stated with *(3) Somewhat applies* (38%, *n* = 42/112), followed by the answer *(4) Fully applies* (32%, *n* = 36/112). Comparing the regular and the reformed curriculum, 73% (*n* = 24/33) of the students in the regular and 55% (*n* = 41/74) in the reformed study program strongly agreed with a SOFA score > 2 as a diagnostic criterion for sepsis (Additional file 1: Fig. S1). Likewise, 78% (*n* = 29/37) of the students of the regular and 75% (*n* = 56/75) of the reformed curriculum did not consider SOFA irrelevant for the diagnosis of sepsis. Testing for differences between the two medical curricula did not yield significant differences (Mann-Whitney *U* test; *p* = 0.08). Ninety-nine percent (*n* = 64/65) of the participants who fully agreed that a SOFA score ≥ 2 was a relevant criterion for diagnosing sepsis also considered a SOFA score ≥ 2 a CT indication. The majority of participants (76%, *n* = 49/64) saw a SOFA score ≥ 2 as an indication for a CT scan (Table 2). Overall, 34% (*n* = 34/100) fully or somewhat considered a SIRS score ≥ 2 to be a diagnostic criterion for sepsis. Conversely, 37% (*n* = 37/100) of participants fully disagreed. When asked whether a SIRS score ≥ 2 can be seen as an indication for requesting a CT in a septic patient, 43% (*n* = 48/112) of students agreed, and 47% (*n* = 53/112) disagreed. Out of the students that chose “does not apply,” 61% (*n* = 22/36) would also refuse to indicate a CT scan based on a SIRS score ≥ 2 (Table 2).

**Table 2:** Results for the categories “clinical criteria for the diagnosis of sepsis” and “the following clinical criteria are an indication for CT.” Only responses from participants who completed both required categories were counted. Data presented in absolute numbers and percentages

	**Clinical criteria for the diagnosis of sepsis**		**Does not apply**		**Rather does not apply**		**Somewhat applies**		**Fully applies**		**I do not know**		**Total**
			***n***	**%**	***n***	**%**	***n***	**%**	***n***	**%**	***n***	**%**	***n***
**The following clinical criteria are an indication for CT**	SOFA score ≥ 2 is relevant in diagnosing sepsis	*Does not apply*	3	33	3	33	3	33	0	0	0	0	9
		*Rather does not apply*	0	0	1	33	1	33	1	33	0	0	3
		*Somewhat applies*	2	8	5	19	13	50	4	15	2	8	26
		*Fully applies*	3	5	9	14	20	31	29	45	3	5	64
		*I do not know*	1	25	1	25	1	25	1	25	0	0	4
	Total		9	8	19	18	38	36	35	33	5	5	106
	SIRS score ≥ 2 is considered a diagnostic criterion for sepsis	*Does not apply*	12	33	10	28	10	28	2	6	2	6	36
		*Rather does not apply*	2	11	10	53	4	21	1	5	2	11	19
		*Somewhat applies*	1	4	9	39	11	48	2	9	0	0	23
		*Fully applies*	0	0	1	9	6	55	3	27	1	9	11
		*I do not know*	1	10	2	20	1	10	1	10	5	50	10
	Total		16	16	32	32	32	32	9	9	10	10	99

*CT* Computed tomography, *SOFA* Systemic Organ Failure Assessment, *SIRS* Systemic inflammatory response syndrome

A majority of 76% (*n* = 49/64) students stated that a SOFA score ≥ 2 is a CT indication, i.e., *(4) Fully applies* and *(3) Somewhat applies* taken together. A third of the students (*n* = 34/100; *(4) Fully applies* and *(3) Somewhat applies* taken together) responding to the question considered a SIRS score ≥ 2 to be a diagnostic criterion for sepsis. Conversely, 37% (*n* = 37/100) of participants fully disagreed. Out of 36 students fully disagreeing with the SIRS score being a criterion for diagnosing sepsis, 6% (*n* = 22/36; *(4) Fully applies* and *(3) Somewhat applies* taken together) would also refuse to establish an indication for a CT scan on the basis of a SIRS score

### Perspective on CT indication in patients with sepsis

**Fig. 3:**
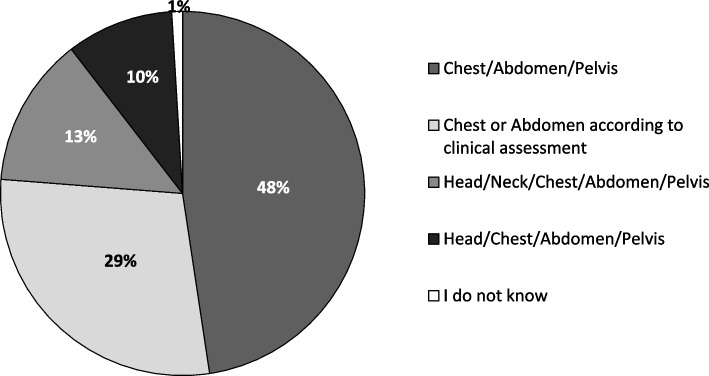
Overview of the body regions chosen by students to be examined by CT to search for a septic focus. The following options were selected in descending order: *chest/abdomen/pelvis* was selected by 48% (*n* = 50/105), *chest or abdomen according to clinical assessment* by 29% (*n* = 30/105), *head/neck/chest/abdomen/pelvis* by 13% (*n* = 14/105), and *head/chest/abdomen/pelvis* by 10% (*n* = 10/105). Total *n* = 105/113; missing responses in 8 participants. CT, computed tomography

Overall, participants saw the confirmation of a suspected focus of infection as one of the advantages of CT in septic patients (83%, *n* = 93/112). The ability to plan interventions and/or surgeries regarding focus control in patients with sepsis was the most notably selected benefit of a CT scan (98%, *n* = 111/113). A large proportion of students did not consider the possible adjustment of anti-infectious therapy after a CT scan as a great benefit (77%, *n* = 86/112), whereas 63% (*n* = 70/112) saw a benefit in CT as a rule-out diagnostic tool (Table 3). When asked to rate procedural options with the information of an initial focus-negative CT scan, most rejected the statement: “If the patient is clinically unaltered, I would like to conduct a repeat CT scan after three days.” On the other hand, the request for a repeat CT scan in cases of clinical deterioration was widely supported (Table 3). Final-year medical students preferred the combined CT examination of the chest, abdomen, and pelvis for focus search in patients with sepsis (48%, *n* = 50/105). A scan of either the chest or the abdomen according to clinical assessment was the second most favored option (Fig. 3).

**Table 3:** Results for indications for repeat CT scans and benefits of CT

		**Does not apply**		**Rather does not apply**		**Somewhat applies**		**Fully applies**		**Total**
		***n***	**%**	***n***	**%**	***n***	**%**	***n***	**%**	***n***
**If the initial CT fails to detect a focus, …**	*and the patient is clinically unaltered, I would opt for a repeat CT scan after 3 days.*	36	33	40	36	26	24	8	7	110
	*I would opt for a repeat CT scan in case of clinical deterioration.*	0	0	10	9	67	60	34	31	111
**I see the greatest benefit of CT…**	*in confirming the suspected diagnosis.*	6	5	13	12	72	64	21	19	112
	*in the modification of anti-infectious therapy.*	45	40	41	37	21	19	5	4	112
	*in planning interventions (e.g., drainage, puncture) and/or surgeries.*	0	0	2	2	44	39	67	59	113
	*in rule-out diagnosis.*	11	10	31	28	57	51	13	12	112

*CT* Computed tomography

Regarding indications for CT, 83% (*n* = 93/112) of the participants saw the advantage of CT in the confirmation of a suspected septic focus *(4) Fully applies* and *(3) Rather does apply* taken together). The ability to plan interventions and/or surgeries for patients with sepsis was selected as a benefit of CT by 98% (*n* = 111/113; *(4) Fully applies* and *(3) Somewhat applies* taken together). Seventy-seven percent (*n* = 86/112) did not regard possible adjustment of anti-infectious therapy with information gathered by a CT scan as a benefit of CT. Sixty-nine percent (*n* = 76/110) rejected the statement “If the patient is clinically unaltered, I would opt for a repeat CT scan after three days” (*(1) Does not apply* and *(2) Does rather not apply* taken together). Ninety-one percent (*n* = 101/111) of participants agreed with the statement “I opt for a repeat CT scan in case of clinical deterioration” for further management after an inconclusive initial CT scan (*(4) Fully applies* and *(3) Rather does apply* taken together)

### Perspective on contrast agent administration

**Fig. 4:**
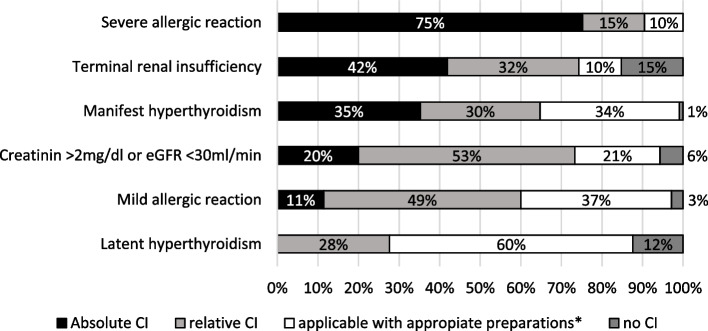
Students’ perspectives regarding the administration of contrast agent for enhanced CT examination of septic patients with additional medical conditions. In 75% (*n* = 79/105), students rated a “known severe allergic reaction to contrast media” as an absolute contraindication to its administration. While 15% (*n* = 16/105) of participants reported no contraindication, 42% (*n* = 44/105) considered a terminal renal insufficiency in a septic patient an absolute contraindication to iodine-based contrast agent administration. For manifest hyperthyroidism, students ticked “no CI,” “relative CI,” and “applicable with preparation” in about 1/3 each, while only 1% (*n* = 1/105) of participants considered it as no contraindication for contrast-enhanced CT. In contrast, 12% (*n* = 13/105) of students saw no contraindication to contrast administration in septic patients with latent hyperthyroidism. Moreover, students did not once tick absolute CI for latent hyperthyroidism. Total *n* = 105; missing responses in 8 participants. CT, computed tomography; eGFR, estimated glomerular filtration rate; CI, contraindication. *Preparation = prophylaxis (including hydration and/or medication) or lower dose of contrast agent

The medical condition classified as the strongest absolute contraindication was a known severe allergic reaction to iodine-based contrast agent (75%, *n* = 79/105) (Fig. 4). For many of the students, a mild allergic reaction in the past indicated a relative contraindication or a necessary preparation before administration of the contrast agent. Terminal renal insufficiency in a septic patient was the medical condition most frequently (15%, *n* = 16/105) classified as no contraindication to iodine-based contrast agent administration. In contrast, it was also the second most common medical condition to be rated as an absolute contraindication (42%, *n* = 44/105). Contrast administration after adequate preparation was most frequently reported in septic patients with latent hyperthyroidism (Fig. 4). Differences in the responses according to experience level as a final-year student were nonsignificant (median = 2.0; *p* = 0.15; Mann-Whitney *U* test; Additional file 1: Fig. S2). None of the students rated more than four of the six listed medical conditions as an absolute contraindication for the administration of an iodine-based contrast agent in septic patients. Of the listed medical conditions, participants with previous radiological experience (*n* = 15/105) selected a maximum of three as absolute contraindications. Seven percent (*n* = 6/90) of students without previous radiological knowledge selected four medical conditions as absolute contraindications. In all three questions regarding the contrast agent administration for the focus search in different body regions, students mostly answered with *(2) rather unenhanced* and (3) *rather intravenous contrast agent* (Table 4).

**Table 4:** Students’ responses regarding the administration of contrast agent in CT examinations of different body regions

	**Unenhanced examination**		**Rather unenhanced examination**		**Rather intravenous contrast agent administration**		**Definitive intravenous contrast agent administration**		**Total**
	***n***	**%**	***n***	**%**	***n***	**%**	***n***	**%**	***n***
**Focus search chest**	13	12	49	45	34	32	12	11	108
**Focus search abdomen**	5	5	28	26	51	47	24	22	108
**Focus search chest/abdomen/pelvis**	7	7	29	27	46	43	25	23	107

*CT* Computed tomography

For a chest CT scan to determine the focus of infection, 57.1% (*n* = 62/108) of the participants chose a *(2) rather unenhanced* or *(1) unenhanced* examination. The answers *(3) rather intravenous contrast agent administration* or *(4) definitive intravenous contrast agent administration* were chosen by 69% (*n* = 75/108) of students for the abdominal focus search using CT. For focus search in the body regions chest, abdomen, and pelvis together, 66% (*n* = 71/107) of the students opted for both *(3) rather intravenous contrast agent administration* and *(4) definitive intravenous contrast agent administration*

### Radiation exposure

Ninety-five percent (*n* = 78/82) of participants answering the question regarding their view on possible negative aspects of CT stated concerns about radiation exposure as an important disadvantage for patients. Radiation exposure was named as a disadvantage of repeated CT scans by 93% (*n* = 14/15) of participants with previous radiology experience versus 96% (*n* = 64/67) of those without such experience. The difference was not significant (*p* = 0.56; chi-square test).

## Discussion

### Summary

Most students reported the SOFA score as a diagnostic criterion of sepsis. A considerable minority opted for the SIRS criteria as diagnostically relevant. The majority of participants who opted for a SOFA sore being relevant for diagnosing sepsis also considered the score’s result as an indication for a CT scan. The ability to plan an intervention or surgery and confirm a suspected focus of sepsis were considered great benefits of CT scans. Students also perceived the benefit of CT as a rule-out diagnostic test. Most students rated a known severe allergic reaction to contrast agents as an absolute contraindication to its administration. Most students were also aware of radiation exposure as an important disadvantage of CT scans.

### Literature

To our knowledge, we present the first survey of final-year medical students’ perspectives on the use of CT in patients with sepsis. Our results partially reflect a lack of knowledge regarding the Surviving Sepsis Campaign (SSC) guidelines among students, as recently reported by Marshall-Brown et al. [13]. A significant minority of our participants considered the SIRS criteria relevant for sepsis diagnosis and, therefore, would rely on outdated diagnostic criteria [19]. Additionally, in the SSC guidelines from 2021, the SIRS score was considered not specific enough and, therefore, not optimal as a single screening tool to identify sepsis [19]. There is an ongoing debate about which score provides the highest accuracy for diagnosing sepsis [19]. In accordance with the Sepsis-3 definition from 2016, most students in our survey assessed the SOFA score as diagnostically relevant [2]. Due to the severity of sepsis, teaching the updated guidelines should be a given. Medical schools need to teach the principles and guidelines, but hospitals where final-year medical students work, should also be responsible for providing current and updated information relevant to treatment. Re-education or re-training on sepsis and CT of students in the later stages of their medical studies may be necessary.

While 65.4% of the participants in the study of Alchallah et al. stated an allergic reaction to contrast media as a contraindication for a CT scan in general, a comparable number of our study’s participants rated a severe allergic reaction to contrast agents as an absolute contraindication but solely for the administration of contrast agents not for a CT scan [20]. In the ESUR guideline on contrast agents, a severe allergic reaction to a contrast agent in the past has been classified as a risk factor, and the use of alternative contrast agents or imaging modalities is recommended [21]. In cases of manifest hyperthyroidism, the guidelines advise against the administration of a contrast agent, whereas only 35% of our study participants classified it as an absolute contraindication [21].

Alchallah et al. described misconceptions about radiation exposure during specific procedures, particularly regarding the dose of a CT scan [20]. Similar results were found by Prezzia et al., who reported poor knowledge about radiation exposure and safety in fourth-year medical students [18]. Conversely, we found a high general awareness of radiation exposure as a disadvantage and risk from CT scans in our student population. Similar to our results, Maharjan et al. assessed the basic level of radiation knowledge as adequate [22]. In contrast to Alchallah et al. and Prezzia et al., we did not ask specific questions about radiation doses and exposure. In compliance with the Radiation Safety Commission’s Guidance on Imaging Studies and the Guidelines for the Referral for Imaging Procedures of the European Union Commission, most participants in our survey favored a CT scan of the trunk, i.e., chest plus abdomen and pelvis, despite the radiation exposure [23, 24]. Besides, based on several studies that localized the most common septic sources to the lungs, abdomen, and genitourinary tract, our group previously showed that these were well identified in CTs of the trunk [10, 11]. De Waele et al. recommend that a CT scan should be considered for focus search in sepsis, especially when there is no clinical improvement [25]. Over half of our participants opted against a repeat CT if the patient’s clinical condition was stable. At the same time, a vast majority would request a repeat CT in case of clinical deterioration. There is an ongoing debate on whether a CT examination should be performed to confirm an obvious focus [25], which is advocated by a minority of students in this survey.

We recently published data from a version of this survey conducted by medical doctors from various disciplines elsewhere [26]. Medical doctors rated the examination of the chest or abdomen according to clinical assessment higher than MD students at 43%. Secondly, the chest, abdomen, and pelvis were opted by medical doctors in 35%. The second part of that survey is currently unpublished.

Essentially, radiology plays a central role in the diagnostic management of patients with sepsis as to identify the infectious focus. All further management will be based on the imaging results, as these are immediately available as opposed to microbiology. Universities and undergraduate studies should account for this central role and adapt curricula to the needs of patient care for junior doctors to be sufficiently trained.

### Limitations

The present study analyzes data from participants of one university clinic and may not reflect the situation at other universities and countries. However, the high proportion of external participants allows conclusions to be drawn about the perspective of final-year medical students who have not only studied at other medical faculties prior to their final year, but also have undergone the regular curriculum teaching. Since parts of the survey’s items were not completed, bias can be assumed. In conjunction with other indicators, leaving blanks seems to reflect uncertainty about the topic of the study among participants. Several participants independently indicated they had no experience and were relatively unfamiliar with certain aspects of the questionnaire. This study focused on self-reported understanding of the use of CT in septic patients and thus did not assess students’ skills at a bedside level or their knowledge of evidence-based guidelines. Due to the limited number of items, the questionnaire may not have been able to capture all aspects of students’ perspectives on the use of CT in sepsis. The results may not represent all medical students as only final-year medical students were surveyed. Due to the small number of students with previous radiological experience in this study, conclusions about the influence of this co-variable carry little weight. Differences between radiology-experienced and radiology-inexperienced students might become evident in a larger number of participants. This study did not analyze the role of other imaging modalities such as ultrasound and X-ray in detail. Further studies should assess students’ knowledge of imaging indications and a stepwise approach to manage patients with infections.

## Conclusion

The majority of participants in our survey displayed familiarity with the sepsis criteria, whereas a minority relied on outdated information. Participants rejected the use of contrast agents in patients with a history of a severe allergic reaction but were less concerned about other contraindications, indicating that more detailed teaching on the potential harm of contrast agents might be beneficial. The greatest advantages of CT in patients with sepsis were seen in the ability to plan interventions and/or surgery based on findings and confirming septic foci. Most participants were aware of radiation as a significant risk of CT. Based on our survey’s results and the feedback the authors received, uncertainty related to CT in sepsis seems common. With its central role in the diagnostic workup of patients with sepsis, radiology should reconsider its undergraduate medical curricula. More attention to the topic of CT in septic patients in the curriculum and possible retraining of students in the practical year could help increase student confidence in this topic.

### Supplementary Information

**Additional file 1:** **Table S1.** Listing of the medical faculties and the medical curricula that the participating final-year medical students had undergone prior to their practical year. **Fig. S1.** Relative frequencies of responses to “SOFA>2 is diagnostic criterion sepsis” in relation to final-year medical students’ study curriculum prior to their practical year. Participants from both study programs selected “fully applies” most often and “somewhat applies” second most often. Thus, 91% (*n* = 30/33) of students from regular and 82% (*n* = 61/74) from the reformed curriculum considered a SOFA score > 2 a diagnostic criterion for sepsis. The answer option “I don’t know” was not once chosen from regular curriculum students, but from 5% (*n* = 4/74) of students from the reformed study program. Total: *n* = 107/113; missing answers in 6 cases. SOFA = Systemic Organ Failure Assessment. **Fig. S2.** Number of medical conditions rated as absolute contraindication for contrast agent administration in septic patients by final-year medical students, sorted by experience in terms of the current trimester. 11% (*n* = 3/27) of first-trimester students, 8% (*n* = 3/37) of second-trimester students, and no third-trimester student selected four absolute contraindications. 15% (*n* = 4/27) of the first, 11% (*n* = 4/37) of the second and 20% (*n* = 8/41) of the third trimester did not classify one of the given medical conditions in septic patients as an absolute contraindication. Most often, first (30%, *n* = 8/27) and second (35%, *n* = 13/37) trimester students indicated 3 of the listed medical conditions as absolute contraindications for contrast agent application, while third trimester (32%, *n* = 13/41) students most often indicated only 2. Total *n* = 105/113; missing responses in 8 participants.

## Data Availability

Further data are provided as supplementary data. Anonymized primary data can be made available upon request to the authors.

## Abbreviations

CI: Contraindication
CT: Computed tomography
SIRS: Systemic inflammatory response syndrome
SIRS score ≥ 2: Greater than or equal to two SIRS criteria
SOFA: Systemic Organ Failure Assessment
SOFA score ≥ 2: SOFA score greater than or equal to two points
SSC: Surviving Sepsis Campaign
