# Supplementary material for: Final-year medical students’ perspective: a survey on the use of computed tomography in sepsis
Source: Insights Imaging. 2023 Nov 19;14:193. doi: 10.1186/s13244-023-01538-y (PMC10657917; doi:10.1186/s13244-023-01538-y)
Supplement: Supplementary file 1 — Additional file 1: Table S1. Listing of the medical faculties and the medical curricula that the participating final-year medical students had undergone prior to their practical year. Fig. S1. Relative frequencies of responses to “SOFA>2 is diagnostic criterion sepsis” in relation to final-year medical students’ study curriculum prior to their practical year. Participants from both study programs selected “fully applies” most often and “somewhat applies” second most often. Thus, 91% (n = 30/33) of students from regular and 82% (n = 61/74) from the reformed curriculum considered a SOFA score > 2 a diagnostic criterion for sepsis. The answer option “I don’t know” was not once chosen from regular curriculum students, but from 5% (n = 4/74) of students from the reformed study program. Total: n = 107/113; missing answers in 6 cases. SOFA = Systemic Organ Failure Assessment. Fig. S2. Number of medical conditions rated as absolute contraindication for contrast agent administration in septic patients by final-year medical students, sorted by experience in terms of the current trimester. 11% (n = 3/27) of first-trimester students, 8% (n = 3/37) of second-trimester students, and no third-trimester student selected four absolute contraindications. 15% (n = 4/27) of the first, 11% (n = 4/37) of the second and 20% (n = 8/41) of the third trimester did not classify one of the given medical conditions in septic patients as an absolute contraindication. Most often, first (30%, n = 8/27) and second (35%, n = 13/37) trimester students indicated 3 of the listed medical conditions as absolute contraindications for contrast agent application, while third trimester (32%, n = 13/41) students most often indicated only 2. Total n = 105/113; missing responses in 8 participants. [file 13244_2023_1538_MOESM1_ESM.docx]

**Final-year medical students’ perspective: a survey on the use of computed tomography in sepsis**

**ELECTRONIC SUPPLEMENTARY MATERIAL**

**Table S1** Listing of the medical faculties and the medical curricula that the participating final-year medical students had undergone prior to their practical year.

| Place of medical studies | N=113 | Study program/medical curriculum |
| --- | --- | --- |
| Charité Berlin | 63 | reformed, practice-oriented |
| Heinrich-Heine-Universität Düsseldorf | 4 |  |
| Universität Hamburg | 3 |  |
| Universität Witten/Herdecke | 2 |  |
| Medizinische Fakultät Mannheim der Universität Heidelberg | 2 |  |
| Medizinische Hochschule Hannover | 1 |  |
| RWTH Aachen | 1 |  |
| Technische Universität München | 6 | traditional, theoretically-based |
| Christian-Albrechts-Universität Kiel | 4 |  |
| Universität Lübeck | 3 |  |
| Eberhard-Karls-Universität Tübingen | 2 |  |
| Friedrich-Schiller-Universität Jena | 2 |  |
| Johann-Wolfgang-Goethe-Universität Frankfurt am Main | 2 |  |
| Justus-Liebig-Universität Gießen | 2 |  |
| Ludwig-Maximilians-Universität München | 2 |  |
| Technische Universität Dresden | 2 |  |
| Westfälische Wilhelms-Universität Münster | 2 |  |
| Albert-Ludwigs-Universität Freiburg | 1 |  |
| Ernst-Moritz-Arndt-Universität Greifswald | 1 |  |
| Friedrich-Alexander-Universität Erlangen-Nürnberg | 1 |  |
| Georg-August-Universität Göttingen | 1 |  |
| Johannes-Gutenberg-Universität Mainz | 1 |  |
| Martin-Luther-Universität Halle-Wittenberg | 1 |  |
| Philipps-Universität Marburg | 1 |  |
| Ruprecht-Karls-Universität Heidelberg | 1 |  |
| Universität Regensburg | 1 |  |
| Universität Rostock | 1 |  |

**Figure S1** Relative frequencies of responses to "SOFA>2 is diagnostic criterion sepsis" in relation to final-year medical students’ study curriculum prior to their practical year.

Participants from both study programs selected "fully applies" most often and "somewhat applies" second most often. Thus, 91% (n=30/33) of students from regular and 82% (n=61/74) from the reformed curriculum considered a SOFA score >2 a diagnostic criterion for sepsis. The answer option “I don’t know” was not once chosen from regular curriculum students, but from 5% (n=4/74) of students from the reformed study program. Total: n=107/113; missing answers in 6 cases.

SOFA = Systemic Organ Failure Assessment

**Figure S2** Number of medical conditions rated as absolute contraindication for contrast agent administration in septic patients by final-year medical students, sorted by experience in terms of the current trimester.

11% (n=3/27) of first-trimester students, 8% (n=3/37) of second-trimester students, and no third-trimester student selected four absolute contraindications. 15% (n=4/27) of the first, 11% (n=4/37) of the second and 20% (n=8/41) of the third trimester did not classify one of the given medical conditions in septic patients as an absolute contraindication. Most often, first (30%, n=8/27) and second (35%, n=13/37) trimester students indicated 3 of the listed medical conditions as absolute contraindications for contrast agent application, while third trimester (32%, n=13/41) students most often indicated only 2.

Total n=105/113; missing responses in 8 participants.
